# Long Non-Coding RNA SNHG6 Supports Glioma Progression Through Upregulation of Notch1, Sox2, and EMT

**DOI:** 10.3389/fcell.2021.707906

**Published:** 2021-08-13

**Authors:** Jing Nie, Yao Feng, He Wang, Xiao-Yu Lian, Ying-Fu Li

**Affiliations:** ^1^Department of Pediatrics, The First Affiliated Hospital of Jiamusi University, Jiamusi, China; ^2^Department of Acupuncture, The First Affiliated Hospital of Jiamusi University, Jiamusi, China; ^3^Department of Neurosurgery, The First Affiliated Hospital of Jiamusi University, Jiamusi, China

**Keywords:** glioma, SNHG6, Notch1, Sox2, EMT

## Abstract

Gliomas, particularly the advanced grade glioblastomas, have poor 5-year survival rates and worse outcomes. lncRNAs and EMT have been extensively studied in gliomas but the disease progression remains poorly understood. SNHG6 has been shown to affect glioma cell proliferation but its effect on EMT of glioma cells along with its effect on disease progression is not known. We screened four glioma cell lines; H4, A172, U87MG, and SW088 and grouped them based on high vs. low SNHG6 expression. Transfections with SNHG6 specific siRNA resulted in induction of apoptosis of high SNHG6 expressing A172 and U87MG cells. This was accompanied by inhibition of EMT and downregulation of EMT-modulating factor Notch1, β-catenin activity and the cancer stem cell marker Sox2. The regulation was not found to be reciprocal as silencing of Notch1 and Sox2 failed to affect SNHG6 levels. The levels of SNHG6 and Notch1 were also found elevated in Grade IV glioma patients (*n* = 4) relative to Grade II glioma patients (*n* = 5). These results identify SNHG6 and Notch1 as valid targets for glioma therapy.

## Introduction

Gliomas account for almost a third of all brain tumors (Goodenberger and Jenkins, 2012). They are particularly aggressive and represent 80% of all malignant brain tumors (Goodenberger and Jenkins, 2012). The median survival of patients with high grade glioblastoma is only about 14 months (Price and Chiocca, 2014) with the 5-year survival almost zero (Stupp et al., 2005; Price and Chiocca, 2014). The median survival of relatively less aggressive grade III gliomas is also dismal; just two to 5 years (Wen and Kesari, 2008; Price and Chiocca, 2014). It is important to study and characterize the etiology and progression of such an aggressive disease, along with elucidation of factors that make it aggressive and could be targeted for therapy.

lncRNAs, the long non-coding RNAs, in recent years, have become a hot topic of research concerning gliomas with hundreds of publications on the topic. A number of lncRNAs have been evaluated for their role in glioma progression (Voce et al., 2019; Yang et al., 2019; Lin et al., 2020; Lulli et al., 2020; Pan et al., 2020; Li Z. et al., 2021). One of the relatively less explored lncRNA in gliomas is SNHG6 with just few reports (Cai et al., 2018; Meng et al., 2018; Zhang et al., 2020; Li X. et al., 2021). This lncRNA can affect glioma tumorigenesis (Li X. et al., 2021). One of the primary mechanisms by which lncRNAs affect tumorigenesis is by sponging microRNAs (miRNAs; Paraskevopoulou and Hatzigeorgiou, 2016; Liao et al., 2020) and SNHG6 has also been reported to sponge several miRNAs, such as miR-101 (Yan et al., 2017; Meng et al., 2018), miR-543 (Zhang et al., 2020; Wang et al., 2021), and miR-944 (Mao et al., 2020), etc. with it being declared as a possible prognostic lncRNA in gliomas (Cai et al., 2018). Despite these evidences for a role of SNHG6 in gliomas, its mechanism of action is not fully understood and, therefore, we performed this study to further evaluate the role of SNHG6 in gliomas. We particularly evaluated the EMT-inducing properties of this oncogenic lncRNA. As modulators of EMT, Notch family members and wnt signaling were evaluated, in addition to the cancer stem cells markers. The results were confirmed in glioma patients derived samples.

## Materials and Methods

### Cell Culture

All the cells used in this study were purchased from ATCC (Manassas, United States) and cultured in DMEM media, with 10% fetal bovine serum, in 5% CO_2_–humidified atmosphere at 37∘C. The cell lines were periodically authenticated in the Genomics core facility.

### Patients

All patients were enrolled at Jiamusi University Hospitals and the archived tissues were used for evaluations. The study was approved by the Ethics Committee at the Jiamusi University (Approval # 20/11-672). Informed consent was obtained from all patients prior to the collection of samples. The investigating team had no access to patient identification data.

### Apoptosis Assay

Induction of apoptosis was assayed using APOSTRAND^TM^ ELISA apoptosis detection kit (Enzo Life Sciences, United States). It is a highly sensitive assay that can detect apoptosis in as little as 500 cells. The assay is based on the sensitivity of DNA in apoptotic cells to formamide denaturation and the detection of the denatured DNA with a monoclonal antibody to single-stranded DNA. For the assay, cells were seeded in 96 well plates, fixed for 30 min, attached to wells by drying for 20 min, treated with formaldehyde for 10 min, denatured for 35 min, blocked, incubated with antibody for 30 min, washed, incubated with peroxide substrate and read at 405 nm, exactly as per the suggested protocol.

### β-Catenin Assay

β*-Catenin* was quantitated using the β-catenin ELISA kit purchased from Enzo Life Sciences, United States. The assay is extremely sensitive that can detect less than 33.8 pg/mL β-catenin. Assay was done in 96 well plates by adding samples directly to the plate, swirling it briefly to mix, sealing to secure contents and shaking at 500 rpm for 1 h. Washings, addition of antibody and further washings and addition of substrate was done, as per instructions. Finally, the readings were done at 450 nm.

### SNHG6 Detection, si-RNA Reagents and Transfections

SNHG6 was detected by primers and detection reagents purchased from Qiagen (China), through the use of qRT-PCR, in patient samples as well as the cell lines. siRNA against SNHG6 was a kind gift from Prof. Wang at University of Jilin, China. siRNAs and controls were purchased from Sant Cruz Biotechnology (United States). All transfections were performed using Lipofectamine 3000 (Thermo Fisher Scientific, China), using standard protocol supplied by vendor.

### Statistics

*p* values were calculated using Student *t* test or one way ANOVA, through GraphPad software. Cell line based studies were conducted a minimum of three times with at least triplicate samples. *p* values less than or equal to 0.05 were deemed statistically significant.

## Results

### SNHG6 Expression in Glioma Cells Lines

**FIGURE 1:**
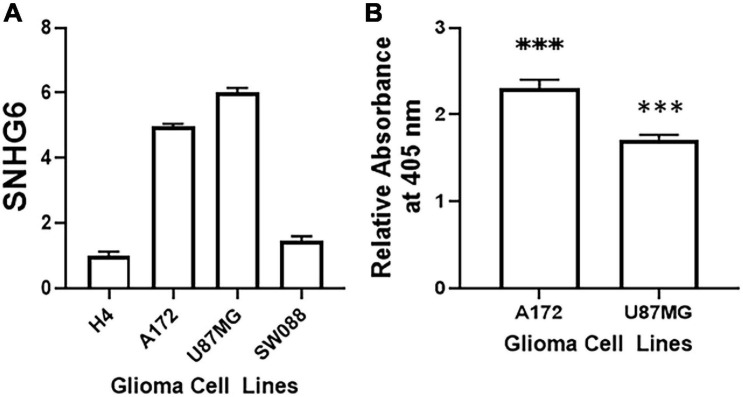
SNHG6 levels in glioma cell lines and effect of downregulating SNHG6 on induction of apoptosis. **(A)** Levels of SNHG6 were evaluated in four glioma cell lines for initial screening by qRT-PCR. **(B)** Induction of apoptosis, upon transfection of SNHG6 siRNA in indicated cell lines, was evaluated using the apoptosis kit described in Methods. The relative OD values are plotted, which are fold-changes compared to the respective cells transfected with non-specific siRNAs. ^∗∗∗^*p* < 0.01.

Our investigation started with an evaluation of the endogenous expression of SNHG6 in some commonly tested glioma cell lines. Specifically, we checked the expression of SNHG6 in H4, A172, U87MG and SW088 cells. As seen in Figure 1A, the four tested glioma cell lines had different levels of SNHG6 with two cell lines, A172 and U87MG expressing high levels of this lncRNA while the remaining two cell lines, H4 and SW088 expressing relatively lower levels of SNHG6. We next evaluated the significance of SNHG6 expression in glioma cells and down-regulated this lncRNA through the use of specific siRNA in two cell lines with the high expression. Downregulation of SNHG6 in glioma cells has been reported to result in induction of apoptosis (Meng et al., 2018). We, therefore, measured induction of apoptosis in A172 and U87MG cells after silencing of SNHG6. As seen in Figure 1B, silencing of SNHG6 resulted in significant induction of apoptosis.

### EMT and Related Pathways in Glioma Cell Lines

**FIGURE 2:**
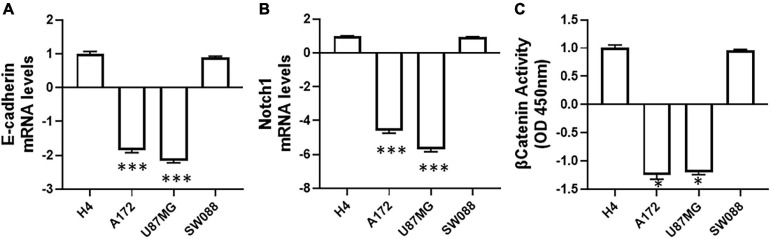
Endogenous EMT and other related markers in glioma cell lines. Levels of **(A)** E-cadherin, the EMT biomarker and **(B)** Notch1 were evaluated in four glioma cell lines by qRT-PCR. GAPDH was evaluated as internal control. **(C)** The activity of β-catenin was evaluated using a commercial kit as described in Methods. ^∗^*p* < 0.05 and ^∗∗∗^*p* < 0.01.

In cancer-related studies, SNHG6 has been connected with the process of EMT (Yan et al., 2017; Wang et al., 2019, 2021; Mao et al., 2020). This is also true for gliomas with at least one such report on the subject (Meng et al., 2018). Thus EMT-regulation seems to be an important process that is regulated by SNHG6. With this information in mind, we decided to evaluate EMT in our study. We compared the four tested lines for their relative EMT status and the expression of key molecules that influence EMT. As seen in Figure 2A, the levels of EMT biomarker E-cadherin were down-regulated ∼two-folds in A172 and U87MG cells, compared to the levels in reference cell line H4.

In human cancers in general as well specifically in gliomas, both Notch1 and β-catenin are connected with the process of EMT (Chen et al., 2016; Li et al., 2018; Zhu H. et al., 2020) and therefore, we next evaluated Notch1 and β-catenin in our study. We found that the levels of Notch1 were similarly down-regulated (similar to E-cadherin levels in Figure 2A) in A172 and U87MG cells (Figure 2B) while the β-catenin activity was also reduced although not to the same extent as Notch1 (Figure 2C). The reduction of Notch1 levels were five to six folds higher. The levels of E-cadherin and Notch1 as well as the activity of β-catenin in SW088 cells were very similar to H4 cells.

### SNHG6 and EMT

**FIGURE 3:**
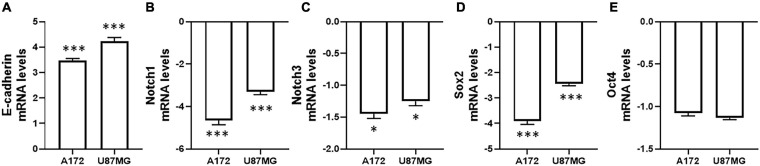
Effect of SNHG6 downregulation on EMT and other related markers. Levels of **(A)** E-cadherin, **(B)** Notch1, **(C)** Notch3, **(D)** Sox2, and **(E)** Oct4 were evaluated in two glioma cell lines, as indicated, by qRT-PCR. GAPDH was evaluated as internal control. The relative values are plotted, which are fold-changes compared to the respective cells transfected with non-specific siRNAs. ^∗^*p* < 0.05 and ^∗∗∗^*p* < 0.01.

We next correlated SNHG6 levels with EMT and associated pathways. In the A172 and U87MG cells that were transfected with siRNA against SNHG6, we first evaluated the levels of EMT marker E-cadherin. As seen in Figure 3A, compared to cells transfected with non-specific siRNA controls, the cells transfected with siRNA against SNHG6 had significantly increased E-cadherin, which is indicative of inhibition of EMT. The transcription levels of Notch1 were also significantly down-regulated in both the cells tested, upon silencing of SNHG6 (Figure 3B). With the implication of Notch3 in the process of EMT as well (Matsuura et al., 2021), we evaluated mRNA levels of Notch3 in cells transfected with SNHG6. As seen in Figure 3C, Notch3 levels were also down albeit not as significantly as those of Notch1. EMT is also related to cancer stem cell characteristics (Brown et al., 2021). Therefore, we evaluated two known biomarkers of stem cell phenotype, Sox2 and Oct4. In glioma cells A172 as well as U87MG, transfections of SNHG6 siRNA markedly reduced the levels of Sox2 (Figure 3D). The mRNA levels of Oct4 were also slightly reduced but were found to be statistically insignificant (Figure 3E).

### Effect of Notch1 and Sox2 Silencing on SNHG6

**FIGURE 4:**
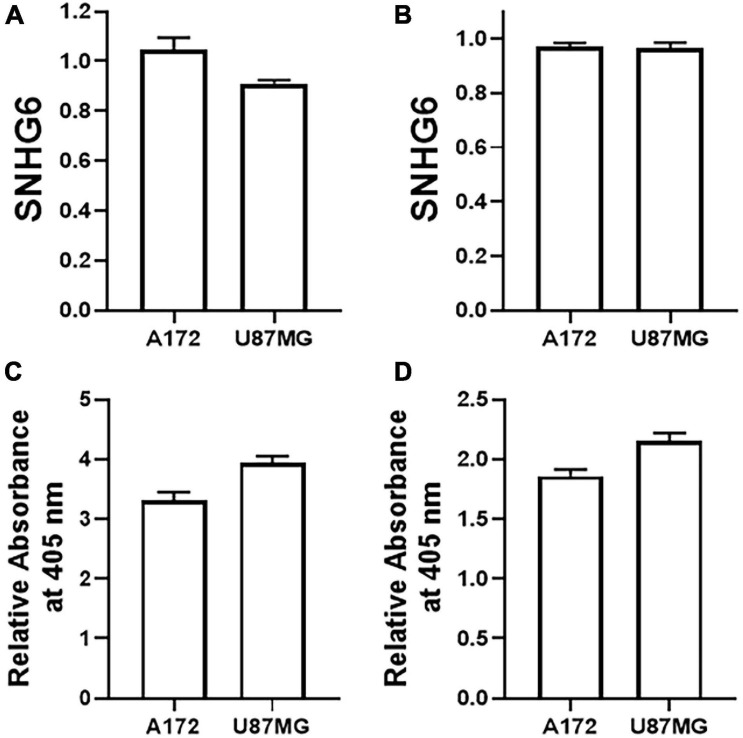
Effect of Notch and Sox2 silencing on SNHG6. Levels of SNHG6 were evaluated in two glioma cell lines, as indicated, by qRT-PCR after silencing of **(A)** Notch1 and **(B)** Sox2. GAPDH was evaluated as internal control. The relative values are plotted, which are fold-changes compared to the respective cells transfected with non-specific siRNAs. Induction of apoptosis, upon transfection of siRNA against **(C)** Notch1 and **(D)** Sox2, in indicated cell lines, was evaluated using the apoptosis kit described in Methods. The relative OD values are plotted, which are fold-changes compared to the respective cells transfected with non-specific siRNAs.

We observed an effect of SNHG6 silencing on expression of Notch1 and Sox 2 in A172 and U87MG cells. To further explore this relationship and to evaluate whether there is bidirectional regulation, i.e., Notch1 and Sox2 can themselves regulate SNHG6 in glioma cells, we silenced Notch1 and Sox2 using specific siRNAs against them and measured SHHG6 levels. As seen in Figure 4A, silencing of Notch1 yielded insignificant results. There seemed to be no effect on SHNG6 in A172 cells while the effect in U87MG cells was very modest and statistically insignificant. Similar results were also obtained when Sox2 was silenced. Again, the levels of SNHG6 were not affected (Figure 4B). We even tested the combined downregulation of Notch1 and Sox2 but still did not see any significant effect on SNHG6 expression (Results not shown). In these cells and under these conditions, i.e., silencing of Notch1 and Sox2, we also measured the induction of apoptosis and observed that silencing of both Notch1 and Sox2 could significantly induce apoptosis. The induction of apoptosis after silencing of Notch1 was much more prominent (Figure 4C), as compared to when Sox2 was silenced (Figure 4D).

### SNHG6 and Notch Levels in Glioma Patients

**FIGURE 5:**
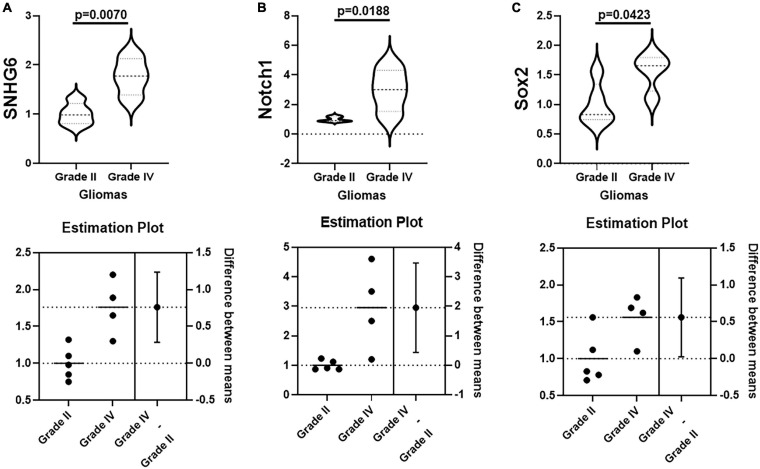
SNHG6, Notch1, and Sox2 levels in glioma patients. Levels of **(A)** SNHG6, **(B)** Notch1, and **(C)** Sox2 were evaluated in patients with Grade II vs. Grade IV glioma, by qRT-PCR. The respective levels in Grade II patients were given a mean value of one and the relative expression levels in Grade IV patients representing fold-changes in the expression levels are plotted. Difference between means were calculated and the significance values are provided.

After our findings in glioma cells lines, we confirmed if the results could hold in glioma patients. For this, we performed a pilot study and evaluated SNHG6 and Notch levels in two groups of glioma patients – patients with Grade II glioma vs. patients with Grade IV glioma. As seen in Figure 5A, SNHG6 was significantly higher in patients with Grade IV glioma with a *p* value of 0.007. At the same time, Notch1 was also significantly higher in patients with Grade IV glioma (Figure 5B). Sox2 levels were also elevated but were barely significant (Figure 5C).

## Discussion

Glioma is the most frequently diagnosed brain tumor with high mortality rate associated with high grade and clinically advanced gliomas (Liang et al., 2020). For our initial experimental setup we first screened a panel of available cell lines in order to investigate the effects of SNHG6 expression. To accomplish this, it was important to list cell lines in order of their differential expression of SNHG6. We observed that cell lines H4 and SWO88 had lower levels of SNHG6 while cell lines A172 and U87MG had higher expression of SNHG6. It is interesting to note that another study studied a similar combination and A172 and U87MG cells were listed as aggressive while H4 and SWO88 were considered less aggressive (Louca et al., 2019). Our findings corroborate this grouping and further highlight the oncogenic nature of lncRNA SNHG6.

In our initial screening of cell lines, we also focused on EMT as EMT plays an important role in glioma progression (Iser et al., 2019; Tao et al., 2020). A number of reports are available in literature that have connected EMT with cell proliferation, invasion and metastasis of gliomas (Zhu H. et al., 2020; Lv et al., 2021). Importance of EMT in prognosis of gliomas has also been suggested (Tao et al., 2020). Another reason for investigating EMT was the reported connection between SNHG6 and EMT (Yan et al., 2017; Wang et al., 2019) which has also been reported in gliomas (Meng et al., 2018). In addition to evaluating E-cadherin, the biomarker for the process of EMT (Loh et al., 2019), we also evaluated Notch1 and the wnt signaling because Notch1 (Qian et al., 2020; Zeng et al., 2020) as well as β-catenin (Liu et al., 2016; Zhang et al., 2018) are intricately connected to induction of EMT in various cancers, including gliomas. While evaluating Notch signaling, we not only evaluated Notch1 but also Notch2, Notch3, and Notch 4. Our observations are indicative of a connection between SNHG6 and Notch 1. Additionally, there seems to be an involvement of Notch3 as well, as seen in our results, even though the effect on Notch1 was much more significant. Interestingly, Notch3 also induces EMT through its interactions with Notch1 (Natsuizaka et al., 2017) and it is possible that SNHG6 might be interacting with multiple Notch family members to induce EMT.

Our results indicate a positive correlation between SNHG6 and induction of EMT. We show an involvement of wnt signaling as well because of the differences we observed in β-catenin upon deregulation of SNHG6. Such involvement of wnt signaling in EMT of glioma cells has been reported recently (Zhu H. et al., 2020). Also, it needs to be acknowledged that similar to our observations with SNHG6, a number of other lncRNAs have also been reported to regulate EMT in gliomas. For example, lncRNA Linc00645 can regulate mesenchymal biomarker ZEB2 and induce EMT in glioma (Li et al., 2019). A role of lncRNAs in even pediatric gliomas’ EMT has been suggested and lncRNA DGCR5 can inhibit EMT in such gliomas where it is down-regulated during the disease progression (Yang and Huang, 2019). Some lncRNAs, such as UCA1 (Li Z.G. et al., 2020), FOXD2-AS1 (Zhao et al., 2020), RP11-84E24.3 (Chang et al., 2021), CTBP1-AS2 (Li Y. et al., 2020), and LINC00525 (Wan et al., 2020) can promote EMT in gliomas while other lncRNAs, such as CASC2 (Wang et al., 2020) and GAS5 (Zhu X.P. et al., 2020) can inhibit EMT in gliomas.

In our study, we observed both the lncRNA SNHG6 and the Notch1 to be elevated. The lncRNAs are frequently reported to sponge miRNAs and those sponged miRNAs have their own gene targets that they inhibit. This relationship results in an inverse relationship between lncRNAs and the miRNAs they sponge but a direct correlation between lncRNAs and the targets of sponged miRNAs because of the de-repression of target genes when miRNAs are sponged. It is possible that the regulatory effect of SNHG6 on Notch1 might involve an intermediate miRNA. We evaluated several potential miRNAs based on published literature as well as bioinformatic analysis but failed to find a miRNA which could functionally fit in this regulatory relationship. Such efforts are still in progress in order to get a more complete picture of regulation of Notch1 by SNHG6. It is interesting to note that we ruled out a reciprocal relationship between Notch1 and SNHG6, at least in our glioma cell line models. Only the silencing of SNHG6 reduced Notch1 and not the *vice versa*. This does not completely rule out the possibility of existence of such regulation and further evaluations might be necessary.

Finally, we confirmed our results using patient samples. We acknowledge the low sample size, however, the aim of this part of the study was to provide a proof of concept. The Grade II tumors we used as one group represent low grade gliomas while the Grade IV tumors in the other group are representative of high grade gliomas. Our evaluation of these two groups further confirms that SNHG6 as well as Notch1 are elevated in high grade gliomas and are thus verified targets for therapy.

## Data Availability Statement

The original contributions presented in the study are included in the article/supplementary material, further inquiries can be directed to the corresponding author/s.

## Ethics Statement

The studies involving human participants were reviewed and approved by Ethics Committee at the Jiamusi University (Approval # 20/11-672). The patients/participants provided their written informed consent to participate in this study.

## Author Contributions

JN and YF performed experiments, evaluated data, and created figures. HW and X-YL evaluated data and created figures. Y-FL supervised study, procured funds, and drafted manuscript. JN, YF, HW, X-YL, and Y-FL edited and approved manuscript. All authors contributed to the article and approved the submitted version.

## Conflict of Interest

The authors declare that the research was conducted in the absence of any commercial or financial relationships that could be construed as a potential conflict of interest.

## Publisher’s Note

All claims expressed in this article are solely those of the authors and do not necessarily represent those of their affiliated organizations, or those of the publisher, the editors and the reviewers. Any product that may be evaluated in this article, or claim that may be made by its manufacturer, is not guaranteed or endorsed by the publisher.
